# Continuous versus intermittent vancomycin in children after cardiac surgery with delayed sternal closure

**DOI:** 10.1186/cc10674

**Published:** 2012-03-20

**Authors:** P Skrak, L Hlinkova, L Kovacikova

**Affiliations:** 1 National Institute of Cardiovascular Diseases, Bratislava, Slovakia

## Introduction

Delayed sternal closure (DSC) is a technique used in patients with hemodynamic instability, lung dysfunction, edema or prolonged bleeding after cardiac surgery. This group of patients has significant morbidity and mortality with fluid overload and changes in renal function. Adequate antibiotic coverage is of great importance and vancomycin is used as a part of antibiotic prophylaxis in our department. The objective of our study was to compare the efficacy and efficiency of intermittent and continuous vancomycin in pediatric cardiac patients with DSC.

## Methods

In a retrospective study we compared three groups of patients: patients with intermittent vancomycin (Intermittent group, *n *= 27) with target trough level of 5 to 10 mg/l, patients with continuous vancomycin (CV1 group, *n *= 24) with target trough level of 20 to 25 mg/l and patients with continuous vancomycin (CV2 group, *n *= 20) with target trough level of 15 to 20 mg/l. The demographic data, total and average vancomycin doses, target level achievement and side effects were analyzed.

## Results

There was no difference in age, weight, surgical complexity and mortality between the groups. The average vancomycin daily dose (mg/kg) was the same in the Intermittent and CV2 groups, the dose was twofold higher in CV1 group (*P *< 0.001) (Table 1). The CV2 group has less trough samples per day of treatment than the CV1 and intermittent groups (*P *= 0.015). Target levels were reached in 42.4%, 30.9%, and 42.9% samples in Intermittent, CV1 and CV2 groups, respectively (*P *< 0.001). Below target were 9.8%, 38.5% and 16.7% samples in Intermittent, CV1 and CV2 groups, respectively. There was no deep sternal infection in any patient. There was similar incidence of peritoneal dialysis in all three groups. No case of renal insufficiency was directly related to increased trough vancomycin level.

**Table 1:** 

	Intermittent group	CV1 group	CV2 group
TLC (*n*)	272	250	233
TLC per day (*n*)	1 (0.6 to 2.3)	0.92 (0.5 to 1.5)	0.81 (0.62 to 1.8)
Daily dose	15 (7.6 to 45)	26.2 (7.6 to 54.3)	15.7 (5.9 to 37.3)

CV, continuous vancomycin; TLC, trough level count.

## Conclusion

In children after cardiac surgery with DSC both intermittent vancomycin with trough level of 5 to 10 mg/l and continuous vancomycin with trough level of 15 to 20 mg/l were comparable with regard to administered dose and target values achievement. There was significantly higher daily dose and trough sample count below target values in patients with continuous vancomycin and target of 20 to 25 mg/l.

